# The Function and Structure of Precuneus Is Associated With Subjective Sleep Quality in Major Depression

**DOI:** 10.3389/fpsyt.2021.831524

**Published:** 2022-02-08

**Authors:** Lu Ma, Cun Zhang

**Affiliations:** ^1^Department of Radiology, Tsinghua University Hospital, Beijing, China; ^2^Department of Radiology, The First Affiliated Hospital of Anhui Medical University, Hefei, China

**Keywords:** major depression, subjective sleep quality, MRI, gray matter volume, neural basis

## Abstract

**Background:**

Poor sleep quality is related to depression. However, the investigation of the neural basis for poor sleep quality in individuals with major depression (MD) is limited.

**Methods:**

Resting state functional and structural MRI data were derived from 114 MD individuals and 74 normal controls (NCs). Fractional amplitude of low-frequency fluctuation (fALFF) and gray matter volume (GMV) were used to measure function and structure of the brain. Pittsburgh Sleep Quality Index (PSQI) was performed to evaluate subjective sleep quality. Correlations were carried out to investigate links of PSQI score with brain imaging indices in MD and NCs, separately. We also examined the differences in fALFF and GMV of brain regions related to PSQI score between MD and NCs.

**Results:**

In contrast to NCs, MD individuals had higher PSQI score. The higher PSQI score was associated with lower fALFF and lower GMV in bilateral precuneus in MD individuals. Moreover, the MD individuals exhibited increased fALFF in bilateral precuneus compared with NCs. However, the correlation between subjective sleep quality and neuroimaging parameters was not significant in NCs.

**Conclusion:**

The implication of these findings is that the function and structure of precuneus provides a neural basis for subjective poor sleep quality in MD. Understanding this may lead to better intervention of depression and associated sleep complaints.

## Introduction

Major depression (MD) is a kind of serious psychiatric condition that limits psychosocial functions and diminishes the life quality (1). Recently, WHO reported that MD is the leading cause of disability worldwide, considering that it influences more than 4.4% of the world's population (2). MD is a clinically heterogeneous illness that is characterized by various symptoms such as depression, anhedonia, sleep complaints, cognitive decline, weight loss, suicidal ideation, and fatigue (1). Sleep complaint is prevalent in MD and more than 90% of individuals with MD (MDs) report sleep problems such as insomnia and early awakening (3). The Diagnostic and Statistical Manual of Mental Disorders (DSM-5) criteria (4) and the International Classification of Diseases (ICD-11) (5) include sleep disturbance in the core symptoms that are present in the diagnosis of depression. Previous efforts have been made to explore the neurobiological mechanisms of the association between depression and sleep disturbance. For example, Santiago et al. reported that changes in cortisol are the modulator between sleep disturbances and depression (6). Lin et al. found 719 shared genes between MD and insomnia, which provide insight into the genetic substrates in depression individuals' comorbid insomnia symptoms (7). Yan et al. proposed that dysfunctional glymphatic pathway may serve as a bridge between sleep disturbance and depression (8). In addition, sleep disturbance has been found to play a vital role in the development of depression (9), an increase risk for depression recurrence (10), poor response to antidepressant treatment (11), and suicidal ideation (12). Therefore, elucidating the neural basis for poor sleep quality in MD helps to understand the neuropathology of disease and develop treatment measures.

Recently, many researchers utilized MRI techniques to examine the neural mechanisms for the associations between sleep and MD and found that the altered function and structure of some brain regions (13–17) and networks (18–20) may serve as the potential neuroimaging biomarkers. Our previous studies tested brain differences between MDs with low and normal sleep efficiency, and further explored associations between sleep efficiency, brain structural and functional alterations, and clinical variables using multi-modal MRI (21, 22). Taken together, these studies not only provided important evidence for the existence of neural basis for poor sleep quality in MD but also validate MRI as an effective tool to investigate this neural mechanism.

In the present study, our goal was to explore the neural basis for poor subjective sleep quality in terms of local neuronal activity and gray matter volume (GMV) in MDs. We used the Pittsburgh Sleep Quality Index (PSQI), the most frequently adopted measure to evaluate subjective sleep quality. Fractional amplitude of low-frequency fluctuations (fALFF) (23), an index assessing the low-frequency oscillation magnitude of blood oxygen level dependent (BOLD) signal, was employed to test the strength of local neuronal activity. We first compared the between-group difference in PSQI score. Next, we explored the correlations of PSQI score with fALFF and GMV in MDs and normal controls (NCs), separately. In addition, inter-group differences of fALFF and GMV in brain regions related to PSQI score were also compared.

## Materials and Methods

### Participants

Data were derived from 114 MD individuals in the Affiliated Psychological Hospital of Anhui Medical University. Two clinical psychiatrists with abundant clinical experience determined the diagnoses according to ICD-10. NCs came from local community and were screened by the Mini-Mental State Examination to exclude any psychiatric disease. For all subjects, exclusion criteria included (1) the presence of other psychiatric illness, (2) a history of major physical or neurological illnesses, (3) a history of severe head injury leading to consciousness loss, and (4) MRI contraindications. For NCs, additional exclusion criteria were a history of psychiatric disorder and first-degree relatives who had a history of psychiatric illness. Hamilton Rating Scale for Depression (HAMD) (24) with 24 items and Hamilton Rating Scale for Anxiety (HAMA) (25) with 14 items were conducted to measure the severity of clinical symptoms including depression and anxiety. Subjective sleep quality was evaluated by using PSQI. All individuals with MD received standard antidepressant medications. The ethics committee of The First Affiliated Hospital of Anhui Medical University approved the study design. All the individuals signed the informed consent before participation. Differences in demographic variables (e.g., age and educational years), brain imaging indices [e.g., frame-wise displacement (FD) and total intracranial volume (TIV)], and clinical variables (e.g., HAMD, HAMA, and PSQI) between the MD and NCs were tested by use of independent sample *t*-tests. Inter-group difference in sex was assessed using Pearson χ^2^ test. The statistical tests were conducted using SPSS 22.0 software.

### MRI Data Acquisition

We obtained MRI scans on a 3.0T MR scanner (Discovery MR750w, GE, Milwaukee, WI, USA) using a comfortable 24-channel head coil. Foam padding and earplugs were utilized to minimize head movement and noise, respectively. All the subjects were asked to keep their eyes closed, hold still, keep awake, and not think about any specific things during the scanning. rs-fMRI data were obtained with a gradient echo single shot echo planar imaging (GRE-SS-EPI) sequence. The parameters for the scan were as follows: repetition time (TR) = 2,000 ms, echo time (TE) = 30 ms, field of view (FOV) = 220 × 220 mm^2^, flip angle (FA) = 90°, matrix = 64 × 64, slice thickness = 3 mm, slice gap = 1 mm, 35 interleaved axial slices, 185 volumes, acquisition time = 370 s. A brain volume (BRAVO) sequence was used to acquire high-resolution three-dimensional T1-weighted structural images: TR = 8.5 ms, TE = 3.2 ms, inversion time (TI) = 450 ms, FOV = 256 × 256 mm, FA = 12°, matrix = 256 × 256, slice thickness = 1 mm, slice gap = 0, 188 sagittal slices, acquisition time = 296 s.

### FMRI Data Pre-processing

Resting-state MRI data were preprocessed with the Data Processing Assistant for Resting-State fMRI (DPARSF, http://rfmri.org/dpabi) (26) based on the Statistical Parametric Mapping (SPM12, http://www.fil.ion.ucl.ac.uk/spm) software. The first 10 time points for each individual were removed to enable the signal to attain equilibrium and the participants to adapt to the noisy scanning. The remaining 175 time points were adjusted for temporal differences across slices. For each volume, head movement metrics were measured by calculating the angular rotation on each axis and the displacement in each direction. Each subject's functional data were constrained within the predefined thresholds (i.e., maximum rotational motion parameter <2° and translational motion parameter <2 mm). FD was also calculated to evaluate the volume-to-volume alterations in the position of the head. Some variables of no interest including linear drift, Friston-24 head movement metrics, “bad” time points with FD more than 0.5 mm, and signals from cerebrospinal fluid and white matter were removed from the BOLD data. For spatial normalization, each subject's T1-weighted map was co-registered with the average functional map. Next, these structural maps were parcellated and transformed to the Montreal Neurological Institute (MNI) standard space utilizing the DARTEL tool (27). Lastly, the functional imaging data were transformed to the MNI standard space utilizing these normalization metrics derived from the aforementioned step and resliced into a 3 × 3 × 3 mm cubic voxel size. After spatial transformation, all the functional data were smoothed by use of a Gaussian kernel of 6 × 6 × 6 mm full width at half-maximum (FWHM).

### fALFF Calculation

To calculate fALFF, we transformed each preprocessed voxel's BOLD signal time series to a frequency domain by using a Fast Fourier Transform approach, with the power spectrum obtained. fALFF was calculated as the ratio of the low-frequency (0.01–0.1 Hz) power spectrum to the power spectrum in the whole frequency range (23). For standardization, each voxel's fALFF value was scaled by the average fALFF value of the whole brain. We used multiple regression in the SPM12 software to find voxels in the fALFF images exhibiting a statistically significant correlation with the PSQI score when adjusting for sex, age, educational years, and FD in individuals with MD and NCs, separately. The cluster-level family-wise error (FWE) approach was adopted to perform multiple testing correction, resulting in a cluster-defining threshold of *p* < 0.005 and a FWE-corrected cluster significance level of *p* < 0.05. If statistically significant associations were found for brain areas in either MDs or NCs, we defined these areas as regions of interest (ROIs) and calculated average fALFF values in these ROIs to further test whether significant group differences in the associations were present. Specifically, at the ROI level, partial correlation coefficients between fALFF and PSQI score were first changed into Fisher's *Z* scores and subsequently compared between MDs and NCs.

To examine whether significant differences were present in fALFF values of the brain regions that were related to PSQI score in MDs or HCs, ROI-based independent sample *t*-tests were carried out. The statistical significance threshold was set at *p* < 0.05.

### GMV Calculation

The three-dimensional T1-weighted maps were processed using the VBM8 (http://dbm.neuro.uni-jena.de/vbm.html) software implemented in the SPM8. Initially, all the T1-weighted maps were closely checked to exclude obvious anatomical deficits or artifacts. After the T1-weighted maps were parcellated into three components including gray matter, white matter, and cerebrospinal fluid, gray matter concentration images were spatially transformed into the MNI standard space. Notably, these images were transformed non-linearly with use of the DARTEL method (27) and resliced to a 1.5-mm cubic voxel. The GMV image was derived via multiplying the transformed gray matter concentration image by the non-linear parameters generated from the previously described spatial transformation step. Lastly, the resulting GMV maps were spatially smoothed by the use of a Gaussian kernel of 6 × 6 × 6 mm FWHM. To examine if the GMV of brain regions identified in the fALFF analysis were correlated with subjective sleep quality, we first calculated the GMV values of these brain areas and then performed ROI-based correlation analysis using SPSS software. Pearson's correlation analysis was used to identify the relationship between GMV and the PSQI score when adjusting for sex, age, educational level, and TIV in MDs and NCs, separately. Partial correlation coefficients were changed into Fisher's *Z* scores and then compared between MDs and NCs. In addition, we also compared GMV of brain areas related to the PSQI score between MDs and NCs. The statistical significance threshold was set at *p* < 0.05.

## Results

### Characteristics of MDs and NCs

The demographic data and clinical characteristics of all subjects are listed in Table 1. The MDs and NCs did not significantly differ in age (*t* = 0.915, *p* = 0.362), sex (χ^2^ = 1.855, *p* = 0.173), TIV (*t* = −1.137, *p* = 0.257), and FD (*t* = −1.838, *p* = 0.068). MDs exhibited higher PSQI score (*t* = 11.377, *p* < 0.001), higher HAMD score (*t* = 21.847, *p* < 0.001), higher HAMA score (*t* = 19.591, *p* < 0.001), and lower educational level (*t* = −6.589, *p* < 0.001) than NCs.

**Table 1:** Demographic and clinical characteristics of the sample.

**Characteristics**	**MDs**	**NCs**	**Statistics**	***P*-value**
Number of subjects	114	74		
Age (years)	42.2 ± 11.3	40.6 ± 12.4	*t* = 0.915	0.362^a^
Sex (female/male)	70/44	38/36	χ^2^ = 1.855	0.173^b^
Educational level (years)	9.5 ± 3.5	13.1 ± 3.9	*t* = −6.589	<0.001^a^
PSQI	12.9 ± 4.9	6.1 ± 3.3	*t* = 11.377	<0.001^a^
HAMD	28.6 ± 11.2	3.3 ± 4.2	*t* = 21.847	<0.001^a^
HAMA	19.4 ± 7.3	3.2 ± 3.9	*t* = 19.591	<0.001^a^
FD (mm)	0.13 ± 0.10	0.16 ± 0.10	*t* = −1.838	0.068^a^
TIV (cm^3^)	1428.1 ± 160.5	1451.1 ± 115.9	*t* = −1.137	0.257^a^

*MD, individual with major depression; NC, normal control; PSQI, Pittsburgh Sleep Quality Index; HAMD, Hamilton Rating Scale for Depression; HAMA, Hamilton Rating Scale for Anxiety; FD, frame-wise displacement; TIV, total intracranial volume*.

a *The p-values were obtained by two-sample t-tests*.

b *The p-value was obtained by χ^2^-test*.

### Correlation Between fALFF and PSQI Score

**Figure 1:**
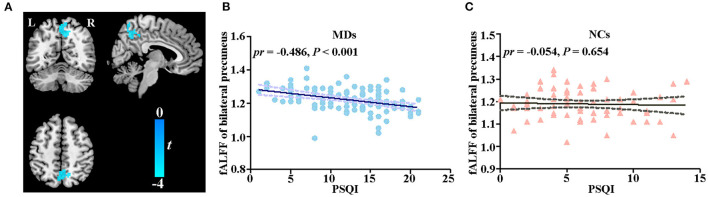
Correlation between fALFF and PSQI score. **(A)** Voxel-based correlation between fALFF of bilateral precuneus and PSQI score in MDs. **(B)** Scatter plot of correlation between fALFF of bilateral precuneus and PSQI score in MDs. **(C)** Scatter plot of correlation between fALFF of bilateral precuneus and PSQI score in NCs. fALFF, fractional amplitude of low-frequency fluctuations; PSQI, Pittsburg Sleep Quality Index; MD, individual with major depression; NC, normal control; L, left; R, right.

In the voxel-wise fALFF analysis, a statistically significant negative association (*p* < 0.05, cluster-level FWE-corrected) was found between the PSQI score and fALFF in the precuneus bilaterally [size of cluster = 194 voxels, MNI coordinate x/y/z of the peak = 12/−42/39, peak *T*-value = −3.9182, partial correlation coefficient (*pr*) = −0.486, *p* < 0.001] after adjusting for sex, age, educational level, and FD in MDs (Figures 1A,B). The correlation between fALFF and PSQI score was not significant in NCs. For completeness, we extracted fALFF of the bilateral precuneus and then conducted ROI-based partial correlation analysis in NCs. The correlation between PSQI score and fALFF was not significant in NCs (*pr* = −0.054, *p* = 0.654, Figure 1C). Moreover, there existed a significant group difference in the PSQI score–fALFF association (*pr*_MDD_ = −0.486, *pr*_HCs_ = −0.054, *Z*-value of group comparison in *pr* = −3.14, *p* = 0.0017).

**Figure 2:**
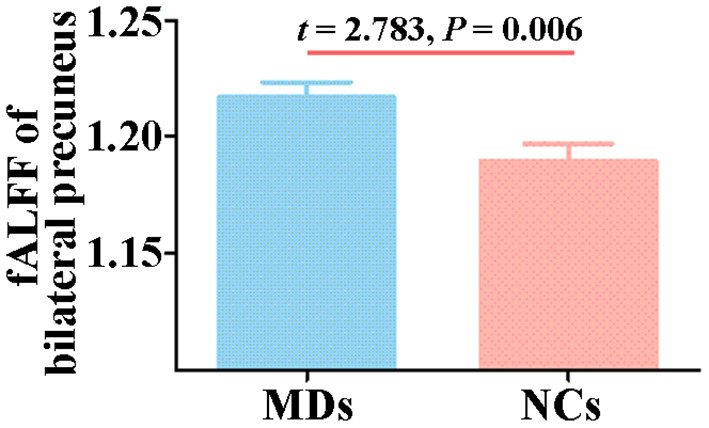
Between-group comparison in fALFF of bilateral precuneus. Error bars indicate the SEM. fALFF, fractional amplitude of low-frequency fluctuations; MD, individual with major depression; NC, normal control.

The group comparison analysis showed a higher fALFF in the bilateral precuneus in MDs (*t* = 2.783, *p* = 0.006, Figure 2).

### Correlation Between GMV and PSQI Score

**Figure 3:**
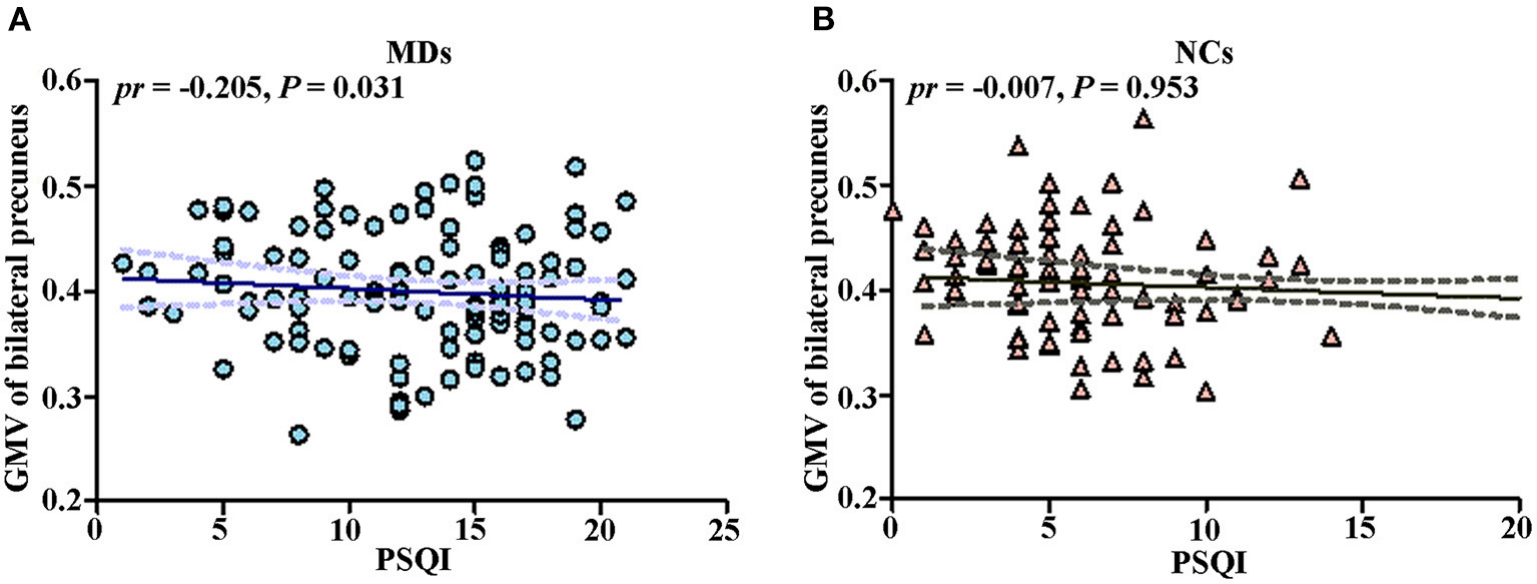
Scatter plots of correlations between GMV of bilateral precuneus and PSQI score. **(A)** Correlation between GMV of bilateral precuneus and PSQI score in MDs. **(B)** Correlation between GMV of bilateral precuneus and PSQI score in NCs. GMV, gray matter volume; PSQI, Pittsburg Sleep Quality Index; MD, individual with major depression; NC, normal control.

To further test whether the GMV of bilateral precuneus was correlated with PSQI score, we performed ROI-based partial correlation analysis between GMV of bilateral precuneus and PSQI score in MDs and NCs, separately. The GMV of bilateral precuneus was significantly associated with PSQI score in MDs (*pr* = −0.205, *p* = 0.031, Figure 3A) rather than in NCs (*pr* = −0.007, *p* = 0.953, Figure 3B). The group difference in the PSQI score–GMV association was not significant (*pr*_MDD_ = −0.205, *pr*_HCs_ = −0.007, *Z* value of group comparison in *pr* = −1.5, *p* = 0.13).

**Figure 4:**
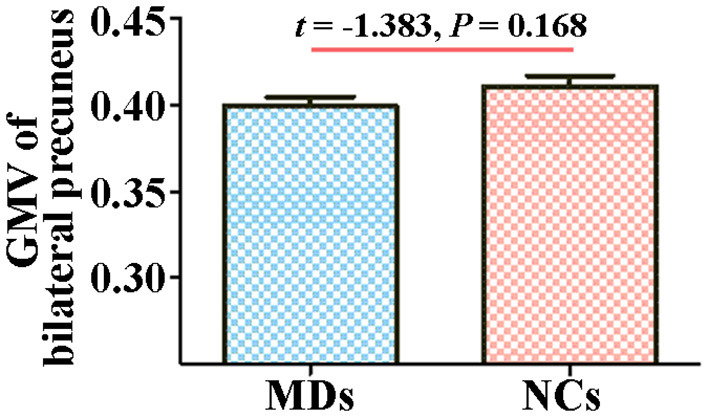
Between-group comparison in GMV of bilateral precuneus. Error bars indicate the SEM. GMV, gray matter volume; MD, individual with major depression; NC, normal control.

There existed no statistically significant difference between MDs and NCs in GMV of bilateral precuneus in group comparison (*t* = −1.383, *p* = 0.168, Figure 4).

## Discussion

Using MRI, we investigated neural basis for poor subjective sleep quality in MDs. We observed three findings in the current study. First, MDs had poorer subjective sleep quality than NCs. Second, in MDs, poorer subjective sleep quality was correlated with lower fALFF and lower GMV in bilateral precuneus. Third, such associations between subjective sleep quality and brain imaging indices obtained in MDs were not present in NCs. Overall, these findings highlight that precuneus may serve as a potential sleep quality–associated neural signature in MDs.

The PSQI is a self-reported questionnaire that evaluates sleep quality over a 1-month time interval (28). PSQI score >5 reflects poor subjective sleep quality. In the present study, the mean PSQI score of MDs is 12.9 and MDs had higher PSQI score compared with NCs. Prior studies have demonstrated that poor sleep quality was seen in MDs (19, 29), which is coincident with our finding.

The precuneus is a key node of the posteromedial cortex, engaging in a wide range of functions, e.g., self-related processing, consciousness, episodic memory, and visuo-spatial imagery (30). The function and structure of precuneus have been proved to be implicated in the neuropathology (31–34) and treatment (35) of MD. Furthermore, precuneus is a central region of the default-mode network, whose impairments have been widely reported in depression (36, 37). The precuneus also plays a pivotal role in sleep disturbance. For example, reduced degree centrality and GMV were found in insomnia patients (38, 39). Functional connectivity and cortical thickness in precuneus were correlated with sleep quality (40, 41). In the current research, PSQI score was negatively correlated with the fALFF and GMV value of the precuneus bilaterally in MDs. These results were congruent with earlier studies. For example, functional connection of the precuneus has been reported to play a mediating role in the relationship between depression and sleep quality in healthy adults (13). In a longitudinal study, the GMV of precuneus was associated with sleep duration via depressive problems in children (42). Collectively, these prior studies, combined with our current finding, suggest that precuneus may serve as the neural basis for poor subjective sleep quality in MD.

In the inter-group comparison analysis, we found that MDs exhibited higher fALFF of bilateral precuneus, suggesting increased local neuronal activity in bilateral precuneus in MDs. This result is different from previous studies investigating the local neuronal activity changes in MD using fALFF method. For example, Li et al. found lower fALFF value of the precuneus in first-episode, drug-naive MDs (43). Liu et al. found decreased fALFF of the precuneus in individuals with recurrent depression. In addition, the decreased fALFF correlated with depression episode numbers (44). These disparities may be linked to the heterogeneity of MDs (MD subtypes, the durations of illness, and antidepression treatments) and sample sizes (32–114 subjects). The absence of inter-group difference in GMV of bilateral precuneus indicated a disassociation between structural and functional alterations. We speculate the reason may be that the structural changes might lag behind the functional changes, which further supports the idea that anatomical structures shape rather than limit regional neuronal activity.

Some limitations should be considered in the current research. First, MDs and NCs were different in educational levels. Even though we regressed educational levels in correlation analysis, the potential effect on result cannot be completely moved. Education-matched NCs are required in future researches. Second, individuals with MD had different illness durations and were receiving antidepressant treatment, which may bias the reliability of the results. Future studies will be conducted in first-episode, drug-naive MDs to confirm the findings. Finally, cross-sectional design is not sufficient for the conclusion of causal relationship. Longitudinal researches are necessary to investigate the causality direction.

In summary, we found that PSQI score was negatively associated with the fALFF and GMV of bilateral precuneus in MDs. Moreover, the fALFF of precuneus was higher in MD compared with NCs. Our findings help to identify sleep quality–associated neural signature and further improve our understanding of the neural basis for poor subjective sleep quality in MD.

## Data Availability Statement

The raw data supporting the conclusions of this article will be made available by the authors, without undue reservation.

## Ethics Statement

The studies involving human participants were reviewed and approved by the Ethics Committee of the First Affiliated Hospital of Anhui Medical University. The patients/participants provided their written informed consent to participate in this study.

## Author Contributions

CZ: conceptualization, methodology, visualization, data curation, writing—review and editing, and supervision. LM: formal analysis, investigation, writing—original draft, visualization, and methodology. All authors contributed to the article and approved the submitted version.

## Conflict of Interest

The authors declare that the research was conducted in the absence of any commercial or financial relationships that could be construed as a potential conflict of interest.

## Publisher's Note

All claims expressed in this article are solely those of the authors and do not necessarily represent those of their affiliated organizations, or those of the publisher, the editors and the reviewers. Any product that may be evaluated in this article, or claim that may be made by its manufacturer, is not guaranteed or endorsed by the publisher.

## References

[1] Malhi GS Mann JJ. Depression. Lancet. (2018) 392:2299–312. 10.1016/s0140-6736(18)31948-2 30396512

[2] Friedrich MJ. Depression is the leading cause of disability around the world. JAMA. (2017) 317:1517. 10.1001/jama.2017.3826 28418490

[3] Tsuno N Besset A Ritchie K. Sleep and depression. J Clin Psychiatry. (2005) 66:1254–269. 10.4088/jcp.v66n1008 16259539

[4] American Psychiatric Association. Diagnostic and Statistical Manual of Mental Disorders. 5th ed. Washington, DC: American Psychiatric Association (2013).

[5] World Health Organization. International Classification of Diseases. 11th ed. Geneva, Switzerland: World Health Organization (2019).

[6] Santiago GTP de Menezes Galvão AC de Almeida RN Mota-Rolim SA Palhano-Fontes F Maia-de-Oliveira JP. Changes in cortisol but not in brain-derived neurotrophic factor modulate the association between sleep disturbances and major depression. Front Behav Neurosci. (2020) 14:44. 10.3389/fnbeh.2020.00044 32410966 PMC7199815

[7] Lin YS Wang CC Chen CY. GWAS meta-analysis reveals shared genes and biological pathways between major depressive disorder and insomnia. Genes. (2021) 12:101506. 10.3390/genes12101506 34680902 PMC8536096

[8] Yan T Qiu Y Yu X Yang L. Glymphatic dysfunction: a bridge between sleep disturbance and mood disorders. Front Psychiatry. (2021) 12:658340. 10.3389/fpsyt.2021.658340 34025481 PMC8138157

[9] Wiebe ST Cassoff J Gruber R. Sleep patterns and the risk for unipolar depression: a review. Nat Sci Sleep. (2012) 4:63–71. 10.2147/nss.S23490 23620679 PMC3630972

[10] Dombrovski AY Mulsant BH Houck PR Mazumdar S Lenze EJ Andreescu C. Residual symptoms and recurrence during maintenance treatment of late-life depression. J Affect Disord. (2007) 103:77–82. 10.1016/j.jad.2007.01.020 17321595 PMC2680091

[11] Franzen PL Buysse DJ. Sleep disturbances and depression: risk relationships for subsequent depression and therapeutic implications. Dialog Clin Neurosci. (2008) 10:473–81. 10.31887/DCNS.2008.10.4/plfranzen 19170404 PMC3108260

[12] Wang X Cheng S Xu H. Systematic review and meta-analysis of the relationship between sleep disorders and suicidal behaviour in patients with depression. BMC Psychiatry. (2019) 19:303. 10.1186/s12888-019-2302-5 31623600 PMC6798511

[13] Cheng W Rolls ET Ruan H Feng J. Functional connectivities in the brain that mediate the association between depressive problems and sleep quality. JAMA Psychiatry. (2018) 75:1052–61. 10.1001/jamapsychiatry.2018.1941 30046833 PMC6233808

[14] Klumpp H Hosseini B Phan KL. Self-reported sleep quality modulates amygdala resting-state functional connectivity in anxiety and depression. Front Psychiatry. (2018) 9:220. 10.3389/fpsyt.2018.00220 29896128 PMC5987592

[15] Gong L Xu R Liu D Zhang C Huang Q Zhang B. Abnormal functional connectivity density in patients with major depressive disorder with comorbid insomnia. J Affect Disord. (2020) 266:417–423. 10.1016/j.jad.2020.01.088 32056908

[16] Wu H Zheng Y Zhan Q Dong J Peng H Zhai J. Covariation between spontaneous neural activity in the insula and affective temperaments is related to sleep disturbance in individuals with major depressive disorder. Psychol Med. (2021) 51:731–40. 10.1017/s0033291719003647 31839025

[17] Yin H Zhang L Li D Xiao L Cheng M. The gray matter volume of the right insula mediates the relationship between symptoms of depression/anxiety and sleep quality among college students. J Health Psychol. (2021) 26:1073–84. 10.1177/1359105319869977 31411064

[18] Liu CH Guo J Lu SL Tang LR Fan J Wang CY. Increased salience network activity in patients with insomnia complaints in major depressive disorder. Front Psychiatry. (2018) 9:93. 10.3389/fpsyt.2018.00093 29615938 PMC5869937

[19] McKinnon AC Hickie IB Scott J Duffy SL Norrie L Terpening Z. Current sleep disturbance in older people with a lifetime history of depression is associated with increased connectivity in the Default Mode Network. J Affect Disord. (2018) 229:85–4. 10.1016/j.jad.2017.12.052 29306697

[20] Gong L Yu S Xu R Liu D Dai X Wang Z. The abnormal reward network associated with insomnia severity and depression in chronic insomnia disorder. Brain Imaging Behav. (2021) 15:1033–42. 10.1007/s11682-020-00310-w 32710331

[21] Yang Y Zhu DM Zhang C Zhang Y Wang C Zhang B. Brain structural and functional alterations specific to low sleep efficiency in major depressive disorder. Front Neurosci. (2020) 14:50. 10.3389/fnins.2020.00050 32082117 PMC7005201

[22] Zhu DM Zhang C Yang Y Zhang Y Zhao W Zhang B. The relationship between sleep efficiency and clinical symptoms is mediated by brain function in major depressive disorder. J Affect Disord. (2020) 266:327–37. 10.1016/j.jad.2020.01.155 32056895

[23] Zou QH Zhu CZ Yang Y Zuo XN Long XY Cao QJ. An improved approach to detection of amplitude of low-frequency fluctuation (ALFF) for resting-state fMRI: fractional ALFF. J Neurosci Methods. (2008) 172:137–41. 10.1016/j.jneumeth.2008.04.012 18501969 PMC3902859

[24] Williams JB. A structured interview guide for the Hamilton Depression Rating Scale. Arch Gen Psychiatry. (1988) 45:742. 3395203 10.1001/archpsyc.1988.01800320058007

[25] Thompson E. Hamilton Rating Scale for Anxiety (HAM-A). Occup Med. (2015) 65:601. 10.1093/occmed/kqv054 26370845

[26] Yan CG Wang XD Zuo XN Zang YF. DPABI: data processing and analysis for (Resting-State) brain imaging. Neuroinformatics. (2016) 14:339–51. 10.1007/s12021-016-9299-4 27075850

[27] Ashburner J. A fast diffeomorphic image registration algorithm. Neuroimage. (2007) 38:95–113. 10.1016/j.neuroimage.2007.07.007 17761438

[28] Buysse DJ Reynolds CF 3rd Monk TH Berman SR Kupfer DJ. The Pittsburgh Sleep Quality Index: a new instrument for psychiatric practice and research. Psychiatry Res. (1989) 28:193–213. 10.1016/0165-1781(89)90047-4 2748771

[29] Lovato N Gradisar M. A meta-analysis and model of the relationship between sleep and depression in adolescents: recommendations for future research and clinical practice. Sleep Med Rev. (2014) 18:521–9. 10.1016/j.smrv.2014.03.006 24857255

[30] Cavanna AE Trimble MR. The precuneus: a review of its functional anatomy and behavioural correlates. Brain. (2006) 129(Pt 3):564–83. 10.1093/brain/awl004 16399806

[31] Lim HK Jung WS Ahn KJ Won WY Hahn C Lee SY. Regional cortical thickness and subcortical volume changes are associated with cognitive impairments in the drug-naive patients with late-onset depression. Neuropsychopharmacology. (2012) 37:838–49. 10.1038/npp.2011.264 22048467 PMC3260976

[32] Crowther A Smoski MJ Minkel J Moore T Gibbs D Petty C. Resting-state connectivity predictors of response to psychotherapy in major depressive disorder. Neuropsychopharmacology. (2015) 40:1659–73. 10.1038/npp.2015.12 25578796 PMC4915248

[33] Zhu J Lin X Lin C Zhuo C Yu Y. Selective functional dysconnectivity of the dorsal-anterior subregion of the precuneus in drug-naive major depressive disorder. J Affect Disord. (2018) 225:676–83. 10.1016/j.jad.2017.08.084 28917194

[34] Yu M Cullen N Linn KA Oathes DJ Seok D Cook PA. Structural brain measures linked to clinical phenotypes in major depression replicate across clinical centres. Mol Psychiatry. (2021) 26:2764–75. 10.1038/s41380-021-01039-8 33589737

[35] Fang J Rong P Hong Y Fan Y Liu J Wang H. Transcutaneous vagus nerve stimulation modulates default mode network in major depressive disorder. Biol Psychiatry. (2016) 79:266–73. 10.1016/j.biopsych.2015.03.025 25963932 PMC4838995

[36] Li B Liu L Friston KJ Shen H Wang L Zeng LL. A treatment-resistant default mode subnetwork in major depression. Biol Psychiatry. (2013) 74:48–54. 10.1016/j.biopsych.2012.11.007 23273724

[37] Sambataro F Wolf ND Pennuto M Vasic N Wolf RC. Revisiting default mode network function in major depression: evidence for disrupted subsystem connectivity. Psychol Med. (2014) 44:2041–51. 10.1017/S0033291713002596 24176176

[38] Altena E Vrenken H Van Der Werf YD van den Heuvel OA Van Someren EJ. Reduced orbitofrontal and parietal gray matter in chronic insomnia: a voxel-based morphometric study. Biol Psychiatry. (2010) 67:182–5. 10.1016/j.biopsych.2009.08.003 19782344

[39] Yan CQ Wang X Huo JW Zhou P Li JL Wang ZY. Abnormal global brain functional connectivity in primary insomnia patients: a resting-state functional MRI study. Front Neurol. (2018) 9:856. 10.3389/fneur.2018.00856 30450072 PMC6224336

[40] Montesino-Goicolea S Valdes-Hernandez PA Hoyos L Woods AJ Cohen R Huo Z. Cortical thickness mediates the association between self-reported pain and sleep quality in community-dwelling older adults. J Pain Res. (2020) 13:2389–2400. 10.2147/JPR.S260611 33061554 PMC7522519

[41] Tian Y Chen X Xu D Yu J Lei X. Connectivity within the default mode network mediates the association between chronotype and sleep quality. J Sleep Res. (2020) 29:e12948. 10.1111/jsr.12948 31793113

[42] Cheng W Rolls E Gong W Du J Zhang J Zhang XY. Sleep duration, brain structure, and psychiatric and cognitive problems in children. Mol Psychiatry. (2021) 26:3992–4003. 10.1038/s41380-020-0663-2 32015467 PMC8855973

[43] Li G Rossbach K Zhang A Liu P Zhang K. Resting-state functional changes in the precuneus within first-episode drug-naive patients with MDD. Neuropsychiatr Dis Treat. (2018) 14:1991–8. 10.2147/NDT.S168060 30122932 PMC6086096

[44] Liu CH Ma X Yuan Z Song LP Jing B Lu HY. Decreased resting-state activity in the precuneus is associated with depressive episodes in recurrent depression. J Clin Psychiatry. (2017) 78:e372–82. 10.4088/JCP.15m10022 28297595

